# Crystallization and Preliminary Analysis of Crystals of the 24-Meric Hemocyanin of the Emperor Scorpion (*Pandinus imperator*)

**DOI:** 10.1371/journal.pone.0032548

**Published:** 2012-03-05

**Authors:** Elmar Jaenicke, Bruno Pairet, Hermann Hartmann, Heinz Decker

**Affiliations:** Institut für Molekulare Biophysik, Johannes Gutenberg-Universität, Mainz, Germany; National Institute for Medical Research, Medical Research Council, London, United Kingdom

## Abstract

Hemocyanins are giant oxygen transport proteins found in the hemolymph of several invertebrate phyla. They constitute giant multimeric molecules whose size range up to that of cell organelles such as ribosomes or even small viruses. Oxygen is reversibly bound by hemocyanins at binuclear copper centers. Subunit interactions within the multisubunit hemocyanin complex lead to diverse allosteric effects such as the highest cooperativity for oxygen binding found in nature. Crystal structures of a native hemocyanin oligomer larger than a hexameric substructure have not been published until now. We report for the first time growth and preliminary analysis of crystals of the 24-meric hemocyanin (M_W_ = 1.8 MDa) of emperor scorpion (*Pandinus imperator*), which diffract to a resolution of 6.5 Å. The crystals are monoclinc with space group C 1 2 1 and cell dimensions *a* = 311.61 Å, *b* = 246.58 Å and *c* = 251.10 Å (α = 90.00°, β = 90.02°, γ = 90.00°). The asymmetric unit contains one molecule of the 24-meric hemocyanin and the solvent content of the crystals is 56%. A preliminary analysis of the hemocyanin structure reveals that emperor scorpion hemocyanin crystallizes in the same oxygenated conformation, which is also present in solution as previously shown by cryo-EM reconstruction and small angle x-ray scattering experiments.

## Introduction

Hemocyanins are giant oxygen transport protein complexes in the hemolymph of invertebrates with molecular masses up to 8 MDa [1]. As such they number in molecular mass among the biggest soluble protein complexes such as cell organelles (e.g. ribosomes) or even small viruses [2], [3]. Due to the enormous size of hemocyanin complexes up to 160 oxygen binding centers can interact with the highest oxygen binding cooperativity (n_H_≥7) observed in nature [4], [5].

All hemocyanins bind dioxygen reversible in μ-η^2^: η^2^ coordination at a type 3 copper active site, which is part of a 4-α-helix-bundle [6], [7], [8], [9], [10]. Depending on the animal group (phylum) two fundamentally different hemocyanin architectures are observed [11], [12], [13]. In molluscs hemocyanins form semi-hollow cylinders with a diameter of approx. 35 nm, which are made up from decamers (M_W_≈4 MDa), didecamers (M_W_≈8 MDa) or multidecamers (M_W_≥12 MDa) of a 350–400 kDa subunit [14], [15], [16]. The molluscan hemocyanin subunit consists of a concatenation of seven to eight paralogous functional units (FU) of approx. 50 kDa each on a single polypeptide chain. Each FU possesses two copper atoms that can bind one dioxygen molecule. The 50 kDa FU is further structurally divided and made up from two or three domains one of which contains the dicopper active site [7], [17]. In contrast, arthropod hemocyanins occur as hexamers (M_W_≈450 kDa) or depending on the species, as oligo-hexamers assembled from several paralogous subunit types with a molecular mass of 75 kDa [18]. Each arthropod subunit is made up from three domains with the middle one containing the dinuclear copper active site. Oligo-hexamers of arthropod hemocyanin are found in distinct aggregation states being two-hexamers (M_W_≈900 kDa), four-hexamers (M_W_≈1.8 MDa), six-hexamers (M_W_≈2.7 MDa) and eight-hexamers (M_W_≈3.6 MDa) [18].

Hemocyanin multimers are generally distinguished by cooperative oxygen binding (homotropic allosteric control) and complex heterotropic allosteric control (Ca^2+^, Mg^2+^, H^+^, urate, lactate, etc) of dioxygen binding. It has been shown that these properties are associated with large conformational changes [19], [20],[21],[22],[23]. Among hemocyanins the four-hexamer hemocyanin of the emperor scorpion (*Pandinus imperator*) is known for its exceptionally high cooperativity with Hill coefficients (n_H_) commonly ranging between 6–7 [24]. Owing to these properties hemocyanins are used as model proteins to better understand their underlying mechanisms both on a functional and structural level. For example a general extension of the two-state MWC model, the Nesting model, was developed and tested using hemocyanins [19], [25], [26]. Furthermore in recent years hemocyanins have gained interest due to the fact that they may serve as ecdysone carriers or act as phenoloxidases after specific activation [27], [28]. As such they can o-hydroxylate monophenols and oxidize diphenols to quinones, which form the black pigment melanin after non enzymatic reaction. The activation of hemocyanins to enzymes can be induced in various ways [28], [29], [30], [31], [32]. In oligo-hexameric arthropod hemocyanins a well characterized conformational change which exposes their active site to the solvent accompanies activation [33], [34], [35].

Structural investigations of arthropod hemocyanins were initiated with the crystal structures of two arthropod hemocyanins, i.e. the native hexamer from spiny lobster and the homohexamer of subunit II from horseshoe crab [8], [9], [36]. Oligo-hexameric hemocyanins have not been suitable for crystallographic analysis until now due to the fact that crystals of oligo-hexamers are difficult to grow most probably owing to the multiple conformational equilibria they manifest.

With the development of cryo-EM reconstruction several low and medium resolution structures of arthropod hemocyanin multimers have been solved in recent years. The cryo-EM structures of *Limulus* (48-mer) and *Scutigera* (36-mer) hemocyanin have been solved at 10 Å resolution [37], [38]. Recently the cryo-EM structure of the emperor scorpion hemocyanin (24-mer) was presented at a resolution of 6.8 Å [33].

However, despite all the advances made with cryo-EM a hemocyanin multimer structure with atomic resolution most probably will only be solved by crystallographic analysis. As part of our endeavor to grow hemocyanin multimer crystals which ultimately diffract to atomic resolution, we present here for the first time the growth and a first analysis of crystals of the 24-meric hemocyanin of emperor scorpion which diffract to 6.5 Å resolution. The molecular mass in the asymmetrical unit of emperor scorpion hemocyanin crystals is one of the largest recorded in the PDB databank comparable to ribosomes, human DNA-dependent protein kinase or erythrocruorin [39], [40], [41].

## Materials and Methods

### Hemocyanin preparation

Emperor scorpions (*Pandinus imperator*) were obtained from “Tropenhaus Hamburg” (Hamburg, Germany). Hemolymph was collected by dorsal puncturing of the pericard and immediately diluted 1∶2 with stabilization buffer [0.1 M Tris/HCl, 10 mM MgCl_2_, 10 mM CaCl_2_, pH 7.8] to prevent coagulation. Since only a few drops of hemolymph could be obtained from one scorpion, it was necessary to pool the hemolymph of several scorpions to obtain a sufficient hemolymph volume for purification. The hemolymph was centrifuged at 4500 g for 10 minutes at 4°C to remove cellular debris. The supernatant containing hemocyanin was applied to a Sephacryl S-300 16/60 HR size exclusion column (GE Healthcare Biosciences, Sweden). The column was eluted with stabilization buffer at a flow rate of 0.6 ml/min) at room temperature. Hemocyanin containing fractions were identified by their absorbance at 340 nm and stored at 4°C. Only fractions containing 24-meric hemocyanin were used for the experiments. The protein concentration of hemocyanin samples was determined by measuring the absorbance at 278 nm using the molar extinction coefficient [ε_278 (nm)_ = 1.04 ml*mg^−1^*cm^−1^] calculated for *Pandinus imperator* hemocyanin using sequence data of the subunits [42]. When necessary, hemocyanin samples were concentrated in Biomax-30K centrifugal filters (Millipore, Schwalbach, Germany).

### Crystallization

Crystals were grown by the “hanging drop” vapor diffusion method in Linbro plates covered with silanized cover slides (Hampton Research, Aliso Viejo, USA). Briefly, 5 µl of a 20 mg/ml hemocyanin solution were mixed with 5 µl of reservoir solution [100 mM Tris/HCl buffer, pH 7.8, containing 2.0% (w/v) PEG [poly(ethylene glycol)] 6000, 1.0 M NaCl, 10 mM CaCl_2_ and 10 mM MgCl_2_] and left to equilibrate in the sealed well at 20°C.

### Data collection and analysis

Prior to data collection the crystals were soaked in mother liquor containing 25% glycerol as cryoprotectant for 30 to 60 seconds. Crystals were then flashed cooled in the gas stream of a cryostream system (Oxford Cryosystems, Oxford, United Kingdom), with a nitrogen gas temperature of 100 K. The crystals diffracted to 6.0 Å resolution on a Microstar rotating anode (Bruker AXS, Karlsruhe, Germany) equipped with HELIOS x-ray optics (Bruker AXS, Karlsruhe, Germany) and a “mar345” image plate detector (MARresearch, Norderstedt, Germany).

Three datasets were collected from the same crystal. For “dataset A” 65 exposures (exposure time: 1200 seconds) were collected for 49° with an increment of 0.75° and a crystal detector distance of 350 mm. For “dataset B” 106 exposures (exposure time: 2400 seconds) were collected for 106° with an increment of 1.00° and a crystal detector distance of 400 mm. For “dataset C” 63 exposures (exposure time: 2400 seconds) were collected for 63° with an increment of 1.00° and a crystal detector distance of 400 mm. Data was processed with the XDS program package (Version: January 30, 2009) and data statistics are shown in Table 1 [43]. The space group was determined using the program POINTLESS (Version 1.4.2) from the CCP4 program suite [44]. For molecular replacement with PHASER a search model of *Pandinus* hemocyanin was built. Briefly, homology models of the different subunits types (2, 3a, 3c, 4, 5a, 5b, 6) were calculated with MODELLER (v9.9) using the structure of a hemocyanin subunit of *Limulus polyphemus* (PDB code: 1NOL) [8], [45]. The model of the 24-meric hemocyanin was obtained by rigid body fitting of the homology models of the respective subunits into the electron density of the resting state of *Pandinus* hemocyanin (EM-databank code: 5100) obtained by Cryo-EM reconstruction [33]. For rigid body fitting of the homology models into the electron density CHIMERA was used [46].

**Table 1:** Crystallographic parameters.

Wavelength	1.54 Å
Space group	*C 1 2 1*
Unit cell	*a* = 311.61 Å, *b* = 246.58 Å, *c* = 251.10 Å
	*α* = 90.00°, *β* = 90.02°, *γ* = 90.00°
Resolution limits	26.8- 6.5 Å (6.7 – 6.5 Å)
Number of unique reflections	36109 (2694)
R_merge_	0.18 (0.76)
I/σ(I)	7.5 (2.0)
Completeness	97.3% (99.2%)
*R* (after PHASER)	0.43

## Results and Discussion

**Figure 1:**
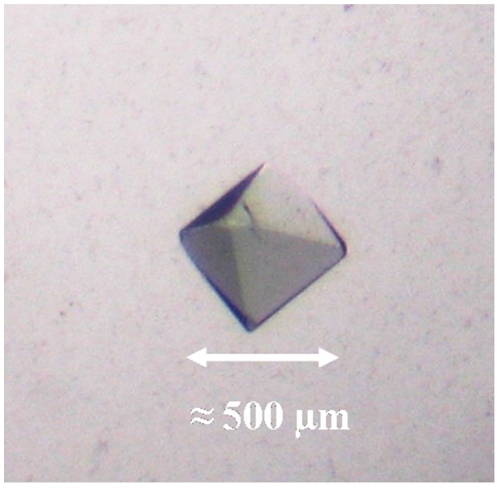
Emperor scorpion hemocyanin crystal. The crystals have the form of small bipyramids and grow to a maximum dimension of 500 µm within weeks after their appearance. The light blue color of the crystals indicated that hemocyanin molecules in the crystal are at least partially oxygenated. This crystal was used to measure the dataset.

**Figure 2:**
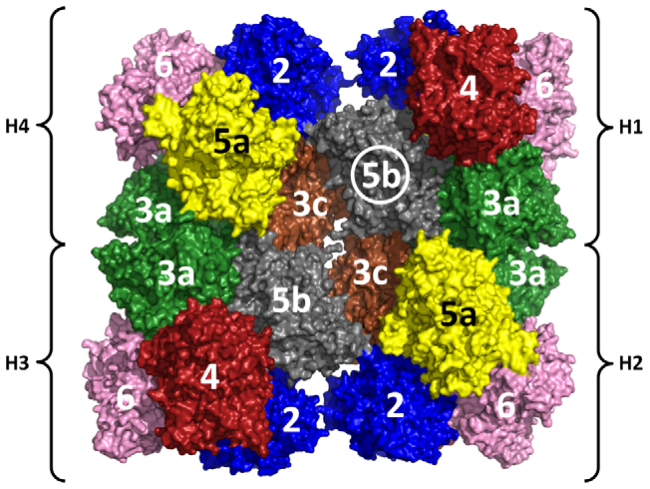
Pseudoatomic model of Emperor scorpion hemocyanin used for molecular replacement. The 24-meric hemocyanin model was obtained by rigid body fit of homology models of the respective subunits into a electron density obtained by cryo-EM reconstruction [33]. For molecular replacement a 12-mer consisting of hexamers H1/H2 was successfully used as search model. Color legend (in brackets the corresponding subunit name in *Eurypelma* hemocyanin is given [47]): SU-3a (Eury-a) = green, SU-5b (Eury-b) = grey, SU-3c (Eury-c) = brown, SU-5a (Eury-d) = yellow, SU-6 (Eury-e) = pink, SU-2 (Eury-f) = blue, SU-4 (Eury-g) = red.

Crystals of the 24-meric hemocyanin of the emperor scorpion (*Pandinus imperator*) were grown by the hanging-drop vapor diffusion method. Small bipyramidal shaped single crystals appeared between three and ten weeks after setup of the experiment. The crystals grew to a maximum dimension of 500 µm within weeks after their appearance (Fig. 1). The light blue color of the crystals indicated that hemocyanin molecules in the crystal are at least partially oxygenated. A complete dataset of a crystal, which diffracted to a resolution of 6.5 Å, was recorded. The crystals are monoclinc with space group C 1 2 1 and cell dimensions *a* = 311.61 Å, *b* = 246.58 Å and *c* = 251.10 Å (α = 90.00°, β = 90.02°, γ = 90.00°). The content of the asymmetric unit was analyzed by molecular replacement. A first analysis suggested that the asymmetric unit contains one copy of the 24-meric hemocyanin with a Matthew's coefficient (V_m_) of 2.79 Å^3^/Da and a solvent content of 56%. For molecular replacement search a pseudoatomic model of the 24-meric hemocyanin taking into account the different subunit types was calculated based on the electron density of a recent 6.8 Å cryo-EM reconstruction [33] (Fig. 2). In order to keep preexisting information about the quaternary structure to a minimum, a first search was conducted searching for four copies of a hexamer cut out from the 24-meric hemocyanin model. However, this search did not produce a meaningful result. Accordingly a second attempt was made by searching for two copies of 12-mer cut out from the 24-mer. This search successfully produced a 24-mer model having the same quaternary structure as the 24-mer from the cryo-EM reconstruction. Thus the molecular mass in the asymmetrical unit of emperor scorpion hemocyanin crystals is one of the largest in the PDB databank comparable to ribosomes, human DNA-dependent protein kinase and erythrocruorin [39], [40], [41].

### Quality of the electron density and model bias

**Figure 3:**
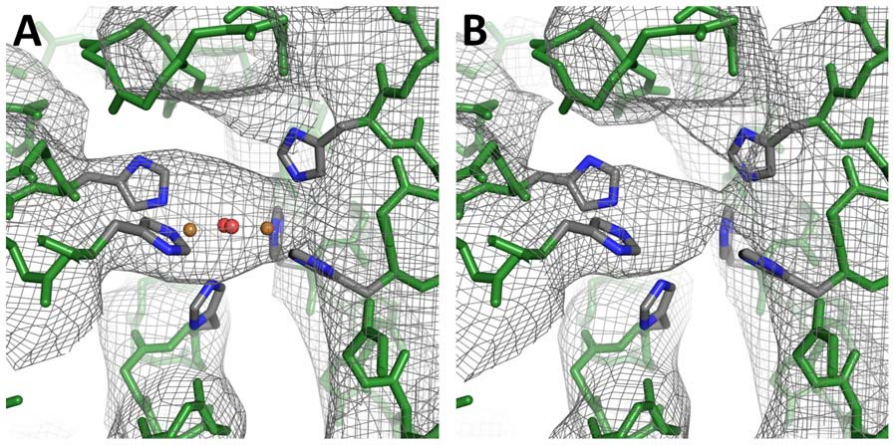
Electron density at the active site. Calculated **e**lectron density (2F_0_-F_C_ map, 1σ contour level) of SU-5b **A**) with all copper and dioxygen atoms included in all 24 subunits of the molecular replacement model, **B**) with all copper and dioxygen atoms omitted in the molecular replacement model. The molecular replacement model included all side chains, but for reason of clarity only the six histidines coordinating the active site are shown. Color coding: backbone atoms = green, carbon = grey, nitrogen = blue, copper = orange, oxygen = red.

The R-factor of the model after molecular replacement was 0.43, which is not unreasonably high taking into account the resolution of the dataset and the fact that no further refinement of the model was made. The quality of the electron density obtained as a result of molecular replacement is very good taking into account its resolution of 6.5 Å (Fig. 3). However, the resulting electron density is likely to be biased by the phases of the search model. A detailed evaluation of model bias as well as refinement of the complete structure are difficult given the enormous size of the protein complex and thus are objective of future work.

In order to get a first estimate of the model bias the electron density was calculated from models, which lacked certain parts. Thus electron density in the spatial volume of the lacking part of the model is not influenced by phase information from the lacking part of the model. This consequently allows within a certain range to distinguish if observed structural features originate from the model or not. In a first trial all active sites were omitted from the model used for calculating phasing. The oxygenated active site of hemocyanin consists of a dioxygen molecule and two copper atoms, which are coordinated by six histidine residues. In the electron density obtained with the 24-meric hemocyanin model obtained from molecular replacement, which includes the oxygenated active site in all 24 subunits, electron density is present in the region, where the active site is expected (Fig. 3A). If in a modified model the copper atoms and dioxygen molecules are deleted in all 24 subunits and electron density is recalculated, electron density is still present in the region normally occupied by copper and dioxygen (Fig. 3B). This indicates that the electron density in the region of the active site is essentially independent from the model input, an excellent indication of the trustworthiness of the model. Nevertheless, the form of the electron density varies to a small extent if the molecules of the active site are present or absent.

**Figure 4:**
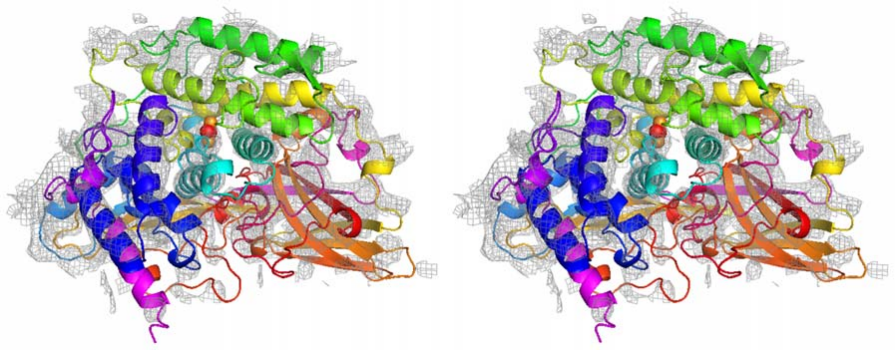
Electron density within the spatial volume of a subunit omitted in the model. One copy of SU-5b (Fig. 2, circled), which forms the central ring of subunits, was omitted from the 24-mer and electron density were calculated from the resulting 23-mer. Electron density, which accounts for secondary structure elements such as α-helices, can clearly be identified in the free space of the omitted subunit 5b.

In a second trial even bigger parts of the model used for phasing were omitted. The hemocyanin of the emperor scorpion is a 24-mer made up from seven paralogous subunit types (Fig. 2). To check the effect of omitting a bigger substructure one copy of subunit 5b (75 kDa), which is located in the center of the hemocyanin molecule, was deleted from the model used for calculating phases. This means that a 23-mer instead of a 24-mer was used for calculating the electron density (Fig. 2, circled subunit 5b). Unexpectedly in the empty space, which formerly was occupied by subunit 5b, almost the complete electron density accounting for α-helices and even that active site could be identified (Fig. 4). This result indicates that model bias in the electron density is not as big as might be suspected, because no preexisting information (i.e. model or non crystallographic symmetry) was used for subunit 5b.

### Hemocyanin conformation in the crystal

The pseudoatomic model of emperor hemocyanin obtained from cryo-EM and the model, which is the result of molecular replacement, are almost identical with respect to their conformation (Table 1). This shows that emperor scorpion hemocyanin crystallizes in the same conformation which is also found in solution (i.e. cryo-EM). The blue color of the crystals confirms that the active sites are oxygenated, which is also suggested by the clearly visible electron density observed in the region of the active site (Fig. 3). Small angle x-ray scattering (SAXS) experiments, which were made previously with the closely related 24-meric hemocyanin of tarantula, also suggest that emperor scorpion hemocyanin in the crystal is in a oxygenated conformation and not in a deoxygenated conformation (Table 2) [20].

**Table 2:** Conformation of the 24-mer hemocyanin molecule.

Method	ϕ_dode_ (°)	d_dode_ (Å)	S_dode_ (Å)
X-ray, MR result	12	110	14
Cryo-EM	13	110	14
SAXS (oxy)	19	104	18
SAXS (deoxy)	6	103	4

ϕ_dode_ = tilt angle between 12-mers; d_dode_ = distance between centers of 12-mers, S_dode_ = distance of parallel shift of 12-mers against each other. SAXS data taken from [20]. Cryo-EM data based on a model constructed from [33].

### Crystal packing

**Figure 5:**
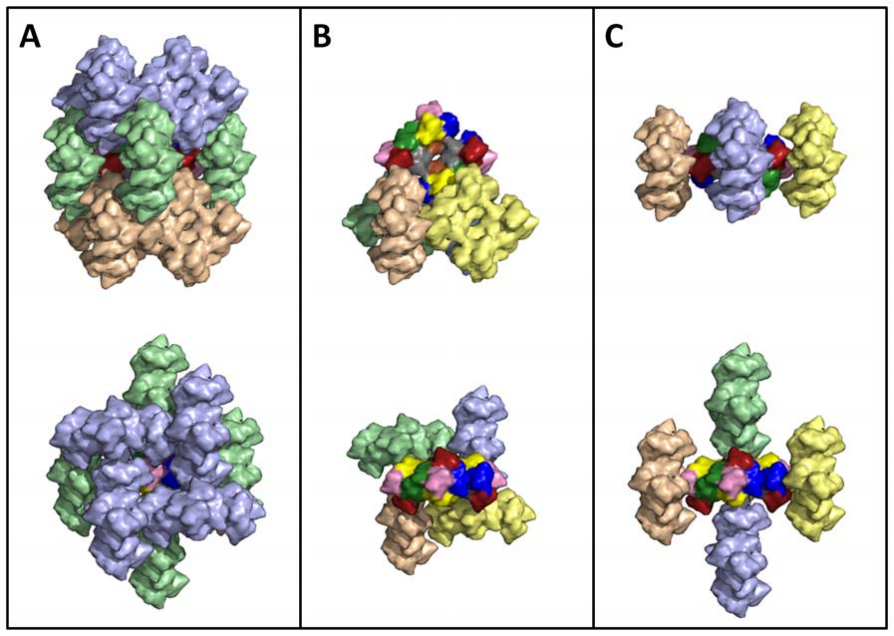
Packing of the hemocyanin crystal. Neighboring 24-mer molecules within a 5 Å distance around a 24-mer hemocyanin molecule are shown. There are 12 next neighbors grouped in an upper, middle and lower ring of four 24-mers each. **A**) Upper ring (light blue), middle ring (pale green) and lower ring (wheat). **B**) The central 24-mer and the lower ring. The spatial arrangement of the upper and lower ring is identical, thus the upper ring is not shown. The four 24-mers of the lower ring are colored in different colors to help in distinguishing them. **C**) The central 24-mer and the middle ring of 24-mers. The four 24-mers of the middle ring are colored in different colors to help in distinguishing them. Upper row: Top views, Lower row: side views.

Despite the enormous size of the 24-meric hemocyanin molecule (20×20×10 nm) the crystals are packed as most other protein crystals and do not contain an above average amount of water (Fig. 5). This is indicated by their Matthews coefficient (2.79 A^3^/Da), which relates to a solvent content of the crystal of 56%. Within the crystal a manifold of different contacts exist between hemocyanin molecules such as for instance an extensive contact region between the outer corner of the 24-mer (subunits 2, 4 and 6) and the central cavity (subunits 3c and 5b) of an adjacent hemocyanin molecule (Fig. 5C). Interestingly an association of two 24-mers in a way similar to the 48-mer hemocyanin molecule of the horseshoe crab (*Limulus polyphemus*) is not observed [38]. We note however that a more detailed analysis of interactions will only be possible when a refined structure is available in the future. These detailed analyses are under way and we anticipate that they will shed important light not only on hemocyanin but also on the structure, assembly and function of organelle-sized macromolecular complexes found in living cells.
